# The Safe Use of Pesticides: A Risk Assessment Procedure for the Enhancement of Occupational Health and Safety (OHS) Management

**DOI:** 10.3390/ijerph16030310

**Published:** 2019-01-23

**Authors:** Mario Fargnoli, Mara Lombardi, Daniele Puri, Laura Casorri, Eva Masciarelli, Stefan Mandić-Rajčević, Claudio Colosio

**Affiliations:** 1 Department of Rural Development, Ministry of Agriculture, Food and Fishery, DISR I, via XX Settembre 20, 00187 Rome, Italy; mario.fargnoli@uniroma1.it; 2 Department of Chemical Engineering Materials Environment (DICMA), Sapienza-University of Rome, via Eudossiana 18, 00184 Rome, Italy; mara.lombardi@uniroma1.it; 3 Department of Technological Innovations and Safety of Plants, Products and Anthropic Settlements (DIT), National Institute for Insurance against Accidents at Work (INAIL), Via Fontana Candida 1, 00078 Monte Porzio Catone (RM), Italy; d.puri@inail.it; 4 Department of Technological Innovations and Safety of Plants, Products and Anthropic Settlements (DIT), National Institute for Insurance against Accidents at Work (INAIL), Via R. Ferruzzi, 38/40, 00143 Rome, Italy; l.casorri@inail.it (L.C.); e.masciarelli@inail.it (E.M.); 5 Department of Health Sciences of the University of Milan, International Centre for Rural Health of the SS. Paolo and Carlo Hospital, Via San Vigilio 43, 20142 Milan, Italy; claudio.colosio@unimi.it

**Keywords:** occupational health and safety, risk assessment, chemical risks, occupational exposure, pesticides, occupational risk management, olive orchards

## Abstract

The attention paid to the use of pesticides has increased notably in recent years as demonstrated by the issue of laws and regulations requiring their safe and environmentally-conscious use (e.g. Directive 2009/128/EC and Regulation (EC) no. 1272/2008). Despite the benefits that can be achieved by pursuing the targets of stricter legislative framework, the difficulties for farmers in complying with it are remarkable, especially for small-sized companies. In fact, in contrast to other occupational health and safety (OHS) contexts, in the case of pesticides even a preliminary analysis on the relationship between pesticide use and the consequent exposure risks for the workers is a complex task. In order to reduce the above-mentioned gap, the present study is focused on the development of an easy-to-use tool for carrying out occupational risk assessment of agricultural activities related to the use of pesticides. The procedure was developed by starting from the Agricultural Health Study (AHS) approach and its improvements, and continuing to the thorough development of a tool for preliminary risk assessment, providing a simplified model for its practical application by farmers. A case study concerning olive cultivation was used for its first verification. The results achieved should be considered as an initial step for the promotion of safer practices when using pesticides, providing a consistent base for their further validation.

## 1. Introduction

In recent years, matters concerning the safer and more sustainable use of chemical products in agriculture have risen in importance worldwide [1,2]. In the European Union this goal is represented by the implementation of the Integrated Pest Management (IPM) policy [3], which relies on a series of legislative interventions starting from the issue of Directive 2009/128/EC, establishing a framework for community action to achieve the sustainable use of pesticides [4]. This directive, together with the related acts affecting, among others, the safety requirements of the machinery for pesticide application [5], the placing of plant protection products on the market [6], the classification, labelling, and packaging of substances and mixtures [7], and the requirements for the registration, evaluation, authorization, and restriction of chemicals [8,9,10], have had a large impact on farmers and companies operating in the agricultural sector.

These new and stricter obligations concern the whole cycle of pesticide use, starting from their purchase up to the disposal of packaging and residues. As requested by Directive 2009/128/EC, the mandatory requirements that should be satisfied for the proper use of pesticides are defined by Member States through their National Action Plans (NAPs) [11]. From the farmers’ perspective, this new approach towards a low pesticide-input in agriculture affects several aspects, ranging from the change of agronomic practices to the compliance with more rigorous specifications for both machinery maintenance as well as occupational health and safety (OHS) measures [12,13,14].

Besides the unquestionable benefits for the environment, human health, and social costs that can be achieved pursuing the targets proposed by this new legislative framework, the difficulties for farmers in complying with it are also remarkable [15,16].

The goal of the present study was to investigate the latter aspect, focusing on the assessment of the occupational risks related to the use of pesticides. Numerous studies addressing risk assessment from the epidemiological point of view can be found in the literature [17,18,19,20,21]. On the other hand, only few studies have dealt with the practical needs of the companies, in particular agricultural enterprises, of implementing risk assessment and management to guarantee the safe use of pesticides [8,22]. In particular, one of the major problems at the practical level, especially for small companies, is represented by the provision of a document where, as per Article 9 of Directive 89/391/EEC, “an assessment of the risks to safety and health at work, including those facing groups of workers exposed to particular risks” is reported. Hence, companies have to implement and document a risk assessment procedure that includes, among other aspects, the best practices, information, and training issues coming from the application of Directive 2009/128/EC, and information provided by the safety data sheet as defined by Regulation (EC) no. 1907/2006 in conjunction with Regulation (EC) no. 1272/2008. Contrary to pesticide exposure, in other types of exposure to particular occupational risks, such as vibrations, noise, or ultraviolet (UV) radiation, it is possible to provide a preliminary evaluation of the exposures and risks. Specifically, when dealing with vibrating tools and machines the exposure levels can be properly defined thanks to the availability of databases recognized from scientific bodies/authorities [23,24]. Based on this, a preliminary risk assessment can be performed considering the dose and the duration of exposure in a hands-on manner [25]. 

In the case of pesticides, the exposure depends on many factors and workers doing apparently the same job might be exposed to different levels of an active substance. There is a lack of clear evidence on how the dose levels or the repeated exposure might influence workers’ health [18,26,27,28]. Moreover, the products put on the market are in continuous evolution [29], and, although some pesticide databases exist [30], their recognition by the OHS authorities is difficult. As a consequence, even a preliminary risk assessment, providing information on the relationships between the pesticides’ use and the consequent exposure risks for the workers, is certainly a complex task [31], requiring additional efforts and resources particularly in the case of small-sized companies [32,33]. In such a context, in fact, safety knowledge, attitudes, and practices are still inadequate [34,35,36], even when dealing with a proper understanding of safety pictograms [37,38,39,40].

In order to reduce the above-mentioned gap, the present study is focused on the development of an easy-to-use tool for carrying out occupational risk assessment of agricultural activities related to the use of pesticides. Such a tool is aimed at supporting companies in verifying the compliance with mandatory requirements and providing OHS management indications to achieve and maintain safe working conditions when using pesticides.

In the next section, an analysis of the legislative framework that the risk assessment activities have to comply with is provided. Then, in Section 3 a background analysis on the scientific issues related to the solution of such a problem is reported. Section 4 presents the research approach/methodology, illustrating the proposed risk assessment procedure, while in Section 5 its practical application in a case study is reported. Section 6 discusses the achieved results and conclusive remarks are presented in Section 7.

## 2. Legislative Framework 

Table 1 synthetizes the interwoven combination of legislative acts, considering their implementation in the Italian legislation and the main issues related to the safe use of pesticides.

From the risk assessment point of view, the combination of the mandatory requisites provided by such legislative frameworks can be translated into the following requirements, which companies have to deal with:

- Provide a documented risk assessment report, demonstrating that the employer has taken into account all the risks derived from the use of pesticides;
- The risk assessment of the activities related with the use of pesticides should consider the worst situation of exposure for each activity;
- All the phases of the process should be considered, starting from handling, preparation of the mixture, setting of the application equipment, mixing, loading, and application, up to the final operations related to the equipment cleaning and maintenance, as well as dealing with the management of the cleaning water and the pesticide residues;
- The risk assessment has to take into account the hazardous properties of the pesticides, the information on health and safety provided by the safety sheet of each product, the level of exposure, and the duration of the exposure, as well as any occupational exposure limit values or biological limit values associated with each product;
- The preventive and protective measure applied, as well as the re-entry safety periods for each type of application have to be defined.

The practical implementation of these provisions requires a high level of expertise and, although a specific training for operators is mandatory to use pesticides, difficulties can arise in correctly understanding and putting them into practice [41]. Moreover, if we consider the Italian context, the companies operating in the agricultural sector are mainly small-sized or family-run enterprises [42,43,44], where the lack of human and financial resources often represents a drawback in properly implementing occupational health and safety duties [45].

To reduce these difficulties, which are similar for small and medium sized enterprises (SMEs) operating in different contexts [46,47,48], a specific easy-to-use risk assessment procedure was developed, which can allow companies to comply with the abovementioned requirements.

## 3. Background Analysis

The risk assessment activities are based on the estimation of the probability of occurrence of a certain event and the severity of its consequences. In the case of chemical risk, such a basic rule can be translated into the evaluation of the toxicity of the chemical product (e.g., based on the acceptable operator exposure level (AOEL) or the “lethal dose” criterion) and the level of exposure of the worker, intended as intensity of exposure per exposure time [49]. The assessment of the exposure to pesticides has been discussed by numerous studies [1,27,29,30,50,51] and different approaches have been proposed, which can be roughly distinguished into: (1) biomonitoring of exposure to pesticides, i.e., the measurement of a pesticide, its metabolite(s), or biotransformation products in biological fluids such as urine or blood [52]; (2) environmental monitoring, consisting in the measure of the exposure in the working environment [53].

Nevertheless, measuring the level of exposure is a difficult and complex task, due to the particularities that characterize the agricultural activities, the differences among the operators and working environments [54]. Accordingly, alternative methods for exposure and risk assessment have been developed, which vary from the use of expert opinion [55,56] and pre-marketing models [57,58] to the use of combination of data from the literature, measurements, and expert opinion [54,59]. In particular, premarketing models were introduced due to the mandatory need for evaluating the exposure of operators as well as for residents and bystanders [60], providing calculators based on databases, such as the BROWSE model [61], EUROPOEM [57], or TOXSWA [62]. Despite their ease of access, these models suffer from several drawbacks when applied in a practical context for occupational risk assessment [63,64]. Similarly, the so-called job exposure matrices (JEMs) [65] present with limited effectiveness when considering the agricultural activities [59,66], and for effective results in the determination of intensity of exposure they should be used in combination with algorithms that take into account other parameters such as the application rate, the type of equipment, and the characteristics of the crop [67].

The latter category includes the widespread Agricultural Health Study (AHS) model [68], developed for the estimation of the exposure to more than 50 individual pesticides, using questionnaire responses and pesticide information published in the literature. Such an approach is considered the most effective means for estimating the intensity level of the exposure to pesticides [69]. Without going into detail since a large literature can be found on the AHS augmentation (e.g., in [18,27,54,70,71,72]), the approach can be summarized in the following equation:

I = (MIX + APPL + REPAIR) × PPE,(1)

where

- I represents the intensity score level, i.e., the exposure level of the operator;
- MIX indicates the exposure during the mixing/preparation activities;
- APPL indicates the exposure during the pesticide application activity;
- REPAIR stands for the exposure during the repair/maintenance/regulation operations of the equipment for pesticide application; and
- PPE refers the personal protective equipment used (e.g., eye glasses, gloves, etc.).

As noted by Mandić-Rajćevič [73], the weights of these factors are based on the monitoring data published in the scientific literature. Although both positive and negative examples of using the AHS algorithm exist in the literature, this system was constructed for epidemiological studies and not for the purposes of the mandatory issues of the OHS legislation. In order to provide a method for the risk assessment of activities related to the use of pesticides, Colosio et al. [54] provided an augmented algorithm based on the AHS approach, which allows a semi-quantitative estimation of the occupational exposure and risk level consequent to pesticide application. Such a study provided practical criteria for the assessment of the exposure factors, which are summarized in Table 2.

Also, modifying factors such as the type of tractor, the type of the PPE used, and the training/skill of the operator were considered. In order to evaluate the toxicity, the criterion used consisted in deriving the toxicity scores based on the risk phrases allocated to the compound of the pesticides used. The advantage of this approach is that it uses the data readily available on the label of the product and does not require any extra training in toxicological evaluation of active substances for the workers. In line with such a framework, other studies (e.g., [74,75]) also provided practical criteria for the estimation of the exposure during re-entry (especially for specific types of farming, such as cultivation in greenhouses), which are based on the density of the plants and the type of pesticide.

Even though the above mentioned studies provide effective guidelines for the assessment of operators’ exposure to pesticides and the related risk levels, they were developed for the purposes of historical exposure assessment in epidemiological studies. Therefore, the models and their various extensions fail to provide a documented risk assessment needed for companies to comply with the health and safety legislation. One example is the option of non-use of proper PPE, which is a legitimate option in epidemiological studies, but is not allowed by the legislation (Directive 89/391/EEC). Additional examples include the specific training of operators as well as the maintenance/calibration of the equipment (Directive 2009/128/EC), which are also cogent requirements.

Additionally, the risk phrases and precautionary statements have been replaced by the “hazard statements” and the related codes due to the entry into force of Regulation (EC) 1272/2008, which would make the approach proposed by Colosio et al. (2012) obsolete [54].

Based on the above considerations, we tried to extend the above-mentioned approaches considering the perspective of an employer that has to draw-up a document where:

1. An assessment of the risks to safety and health at work is reported, including those facing the workers exposed to chemical risks;
2. The protective measures to be taken as well as the personal protective equipment to be used are defined; and
3. The specific training of operators and the related mandatory certificates are listed.

## 4. Materials and Methods 

**Figure 1:**
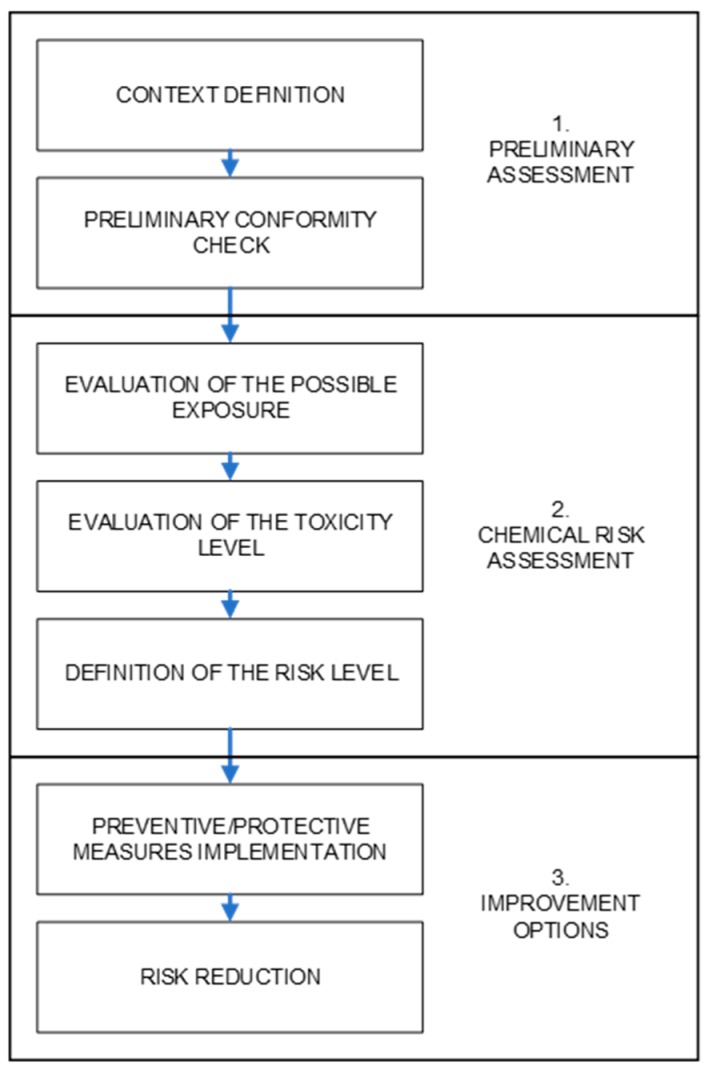
Scheme of the proposed approach.

The proposed approach consists of three main phases (Figure 1). In the first phase, a preliminary check is carried out in order to verify if the company is compliant with general OHS requirements related to the use of pesticides. Then, the specific assessment in case of chemical risks is carried out, evaluating the exposure level of the operator related to the toxicity of the products used. Although the procedure we propose represents a simplified and preliminary assessment of chemical risk, in accordance with the national and EU OHS legislation, we use the locution “chemical risk assessment” for this phase. Finally, the improvement options are proposed for a proper risk management.

### 4.1. Preliminary Assessment

In this phase, a preliminary analysis of the context has to be performed in order to understand the company’s practical needs and the working tasks that should be performed. Accordingly, the employer has to verify the compliance with the general OHS requirements regarding the following topics:

1. Professional information and training of the operator and the related certification as the pesticides’ professional user;
2. Machinery conformity (tractor and pesticide application equipment);
3. Selection of the proper pesticides depending on the company needs;
4. Functional calibration of the application equipment;
5. Definition of working procedures for the pesticides’ application;
6. Selection of the proper PPE based on the type of pesticides and machinery used; and
7. Conformity with the health surveillance requisites.

The fulfilment of all these requirements is mandatory and to support their management a specific checklist was developed (Appendix A). The accomplishment of such requirements provides the necessary input to perform the specific evaluation of the so-called “chemical risks”.

### 4.2. Chemical Risk Assessment

This analysis consists of a preliminary assessment (i.e., before the application of the pesticides is carried out) aimed at the definition of an exposure risk profile, and it is realized through a three-step approach. In the first step, the levels of exposure are estimated, considering all the possible exposure determinants present in the scenario under evaluation. Based on their conditions in this scenario, a numeric weight is assigned, and the obtained values are elaborated through a specific algorithm, to obtain and estimate of the possible level of exposure. Then, a value of toxicity is assigned considering the characteristics of the product that will be used. Finally, the estimation of the risk level is computed combining these values.

#### 4.2.1. Evaluation of the Possible Exposure

To evaluate the possible exposure level (I_exp_) of the operators, the algorithm proposed by Colosio et al. [54] and Mandić-Rajćevič [73] was improved as follows:

I_exp_ = [(MIX × *t_M_* + APPL × *t_A_*+ REPAIR × *t_R_* + RE-ENTRY× *t_RE_*) × SKILL × PPE] × FREQ (2)

where the possible re-entry of the operator was included (RE-ENTRY), as well as a reducing factor considering the experience of the operator (SKILL), while PPE considers the status of the personal protective equipment and FREQ indicates how many times in a year the operator carries out activities involving the use of pesticides. With reference to the latter aspect, it has to be noted that, according to the current Italian OHS legislation, the employer has to update the risk assessment document at least yearly. As far as the time *t* is concerned, *t_M_*, *t_A_*, *t_R_*, and *t_RE_* represent the percentage of time dedicated to the MIX, APPL, REPAIR, and RE-ENTRY activities, respectively in a working day, taking into account that according to the Italian OHS legislation the duration of a working day is of 8 h. In other words, we assumed that the sum of these factors is not more than 100% of time of a regular working day, since the abovementioned activities cannot overlap each other as they are carried out sequentially by one operator only in a working day. Regarding the factor SKILL, the criteria used to introduce this aspect in Equation (2) are based on the information provided by the technical guideline provided by the International Social Security Association (ISSA) [76], where the skills of the operator are estimated considering its experience in carrying out a specific task. 

As far as the RE-ENTRY working activities are concerned, they usually consist in any type of activity performed in a field where pesticides were previously used. From the legislative point of view, in line with the provisions of the Italian NAP, the re-entry in the field is allowed only 24 h after the pesticide’s application. Then, during the following 24 h the operator can enter in the field only with the proper PPE; after that (i.e., after 48 h) the use of the pesticides’ PPE is not mandatory. From an agronomic perspective, it has to be underlined that in some types of cultivations (e.g., maize or rice) the re-entry is not foreseen and thus it should not be computed. In our study, we included in this category the following situation: the operator needs to refill the atomizer’s tank to complete the application and goes back and forward from the loading point to the application area. In case the cultivated field has more than one access, the latter activity is not computed if the operator can use other entry points avoiding the already sprayed areas. Although such criteria might appear quite simple, they are based on the experiences with farmers, which are often oriented towards practices that allow them to save time and resources.

The assessment criteria and the relative scores of the elements of Equation (2) are reported as follows: Table 3 (MIX), Table 4 (APPL), Table 5 (REPAIR), Table 6 (RE-ENTRY), and Table 7 (PPE, SKILL, and FREQ). The criteria used in the following tables are based on multiple sources: the starting point consisted in the findings and the related scientific review provided by [36,64,73]; as additional references for the weighting factors we have considered technical guidelines in the field of pesticide management [77] and from other fields such as mechanical hazards [76]. Moreover, the weighting factors were further discussed in a group of experts in order to verify their usability and effectiveness.

It has to be noted that CONC refers to the concentration of the active principle used in the product: expressed as a percentage: this information can be depicted from the product’s Safety Data Sheet (SDS) and it is expressed as a weight/weight (w/w) percentage (as indicated in Regulation (EC) no. 1272/2008).

It has to be noted that in Table 4, both INT and EQUIP were considered as additional weighting factors of the exposure risk during the application phase. This is because especially in small companies the attention paid to the condition of the application equipment is still limited [78,79,80].

Based on the results obtained by means of equation (2), for each pesticide used it is possible to define an exposure level (I_exp_), which can be classified as depicted in Table 8 following the criteria proposed in [42].

#### 4.2.2. Evaluation of the Toxicity Level

The creation of a grid for the evaluation of the toxicity exposure needs the synthesis of toxicity levels in ranked numeric values. As observed by Maroni et al. [31], despite its qualitative nature, the use of the information provided by the product labels for a preliminary risk assessment in an OHS context can be considered effective. Such an approach can answer to the needs of safety managers or entrepreneurs that can use the data provided by the pesticides’ producers in compliance with the mandatory authorization’s requirements [9], as practical information for a safe management of pesticides. For this reason, as a reference we used the list of substances provided by Regulation (EC) no. 1272/2008, although other types of lists proposed at the international level [81,82] can be found.

Based on this, we defined five levels of toxicity using as a surrogate of the potential toxicity the hazard statements (H) established for toxic substances by the Regulation (EC) no. 1272/2008. The grid is shown in Table 9, where a 1 to 5 toxicity index (I_tox_) is considered (1 = very low; 5 = very high). The use of a Likert scale [83], ranging from 1 (not important) to 5 (extremely important) is quite common in qualitative assessment and its application in risk assessment is foreseen by the ISO/IEC 31010 standard [84].

In the grid both hazard statements (H) and supplemental hazard statements relating to particular physical and health properties (EUH) were included: they were selected considering the pesticides most used in Italy. From the practical standpoint, it is worth noting that most of the products available on the market present more than one hazard statement. Hence, when carrying out the risk assessment, the value given to the product is established based on the highest score of the toxicity index.

#### 4.2.3. Definition of the Risk Level

The estimation of the risk level (R_E_) is performed combining the possible exposure level (I_exp_) and the toxicity index (I_tox_) by means of the following equation:

R_E_ = I_exp_ × I_tox_ (3)

The output of the second phase consists in the classification of the exposure risk level based on the criteria exposed in Table 10: the definition of the different levels’ ranges are based on the suggestion provided by the ISO/IEC 31010 technical standard [84] for the implementation on the risk matrix. 

### 4.3. Improvement Options

Based on the results obtained in the second phase, and the levels of risk pointed out, the next phase of the evaluation consists in the definition of the need of risk management options addressed at reducing the risk to acceptable values. In Table 11 the main preventive interventions which can be done by the company are shown.

Once the preventive/protective measures are put into practice, the level of the exposure risk has to be assessed again in order to verify the levels of risk anticipated after the intervention. It is worth noting that the advantage of this approach is that the risk is assessed before the application, and the application is done only when it has been proved the absence of an unacceptable risk. 

Following the provisions of the OHS legislation, this information has to be documented in a proper risk assessment file concerning each type of pesticide used by the company. If the company uses more than one pesticide, the whole procedure described in the previous sections has to be applied for each product. Hence, the proper preventive/protective interventions have to be defined considering all the information collected, i.e., the overall risk assessment activities described in this section.

## 5. Case Study

**Figure 2:**
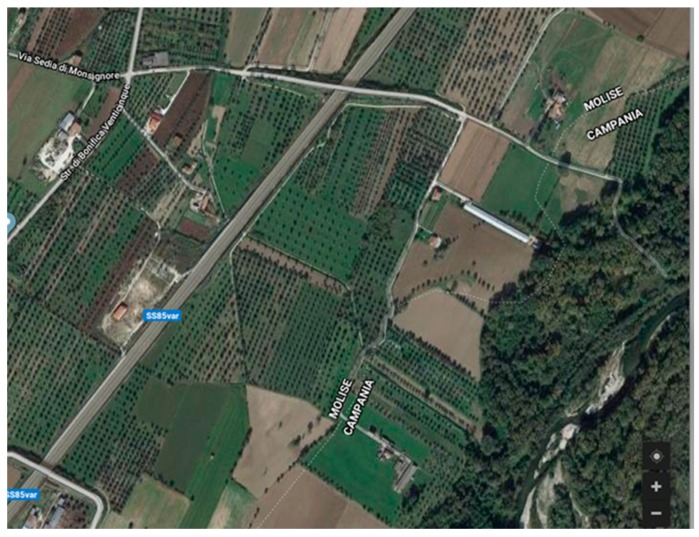
Aerial photo of the field.

In order to validate the proposed approach, we carried out, in collaboration with an Italian company engaged in olive oil production, an “in-field study”. In particular, we considered a field of about 2 hectares, where 210 olive trees are cultivated (Figure 2).

### 5.1. Preliminary Assessment

The typical pathologies that can affect the olive trees and their fruits in the area where the company’s fields are located (middle part of Italy) are the olive fly (*Bactrocera* or *Dacus oleae*) and the so-called “olive peacock spot” (*Spilocaea oleaginea*), which are quite common in olive cultivation [85]. More in detail, considering the number and the dimensions of the trees, as well as the characteristics of the field, the following products are used by the company:

- Product “A” against the “olive fly”: a synthetic pyrethroid is used, whose active principle is based on the presence of deltamethrin (whose concentration derived from the SDS is 1.51 %). As far as the quantity of product is concerned, the use of 0.9 L of pesticide was estimated for 1000 L of water (i.e., the company uses 0.45 L of product A per hectare). The re-entry time is fixed in 3 days after the treatment.
- Product “B” against the “olive peacock spot”: a copper compound is used, i.e., a tribasic copper sulfate (whose concentration derived from the SDS is 24%). The needed quantity was estimated in 3.0 L per 1000 L of water (i.e., the company uses 1.5 L of product B per hectare). The re-entry time is fixed in 20 days after the treatment.

The sprayer used for the application is a tractor-mounted mist blower for medium volume air treatment with a capacity of 500 L. Thus in both cases it has to be used twice. The tractor is equipped with a non air-conditioned cabin. As far as the treatments’ schedule is concerned, both of them are applied in a 1 day working shift. 

All the requisites of the conformity checklist (Appendix A) were verified positively: in particular, the three operators attended the specific training, holding the certificate of competence as professional pesticide users. One of them has 1 year of experience, while the other two have more than 5 years. Hence, in the assessment the case of the first one is considered. The PPE was considered adequate to the type of substances used and resulted in good condition.

### 5.2. Evaluation of the Possible Exposure Level

Two different assessments were done, for each of the two products used by the company. In particular, the evaluation of the possible exposure level related to both the product “A” and “B” are summarized in Table 12. In detail, for both products the scoring related to the considered surface (SURF) is equal to 0.5 (as per values indicated in Table 4), while for the estimation of DOSE we assumed that the specific weight of the formulation is roughly that of water (1 L = 1 kg). Then, following the criteria exposed in Table 4, in both cases the DOSE = 2.

Based on the criteria exposed in Table 8, both the potential exposure levels can be ascribed as belonging the level 1, i.e., “very low level of possible exposure”. It has to be noted that in both cases the re-entry activities were considered only once, when the operator has to refill the sprayer’s tank.

### 5.3. Evaluation of the Toxicity Level

The analysis of the toxicity level of the pesticides used by the company is carried out based on the information provided in their safety sheets (Table 13).

### 5.4. Evaluation of the Risk Level

Combining the possible exposure level and the toxicity level as per Equation (3), the exposure risk level (R_E_) for both products was estimated (Table 14).

### 5.5. Improvement Options

While the risk level when using the product “A” resulted “acceptable” and no further interventions are required, in the case of “B” the risk level is higher mainly due to the limited experience of the younger operator. For this reason, additional training activities were foreseen, and the company decided to assign the application of “B” only to the two more experienced operators. Consequently, the risk index was reduced to R_E_ = 10.18, reaching the level of acceptability. A further improvement could be achieved providing a proper route in the orchard in order to avoid or reduce the exposure time during the re-entry when the refill of the sprayer is needed.

## 6. Discussion

The results achieved were considered positively by the company since our approach allowed them to perform a complete analysis, updating their risk assessment documents in a user-friendly manner. Moreover, the checklist for the preliminary assessment was considered a useful tool for monitoring the proper application of safety procedures.

From a more general perspective, the problem of providing tools that allow the estimation of the risk of the exposure to pesticides, contributing to the definition of exposure limits and to the prevention of the toxic effects on workers is widely discussed in the literature [1,67,81]. As a matter of facts, the risk assessment related to the exposure to pesticides presents numerous variables, making it a more complex task than when dealing with other types of hazards agricultural workers are exposed to [28]. In such a context, the definition of a tool aimed at supporting safety managers in the documented preliminary risk assessment of working activities related to the use of pesticides represents a narrow aspect of the problem, although the relevant impact it might have on the practical needs of companies. In such a research niche, the present study can be considered a first attempt of merging all the mandatory issues related to the use of pesticides in an easy-to-use procedure for the correct implementation of OHS risk assessment activities. 

In particular, our study is based on the results achieved in the epidemiological literature, together with the provisions of technical guidelines and the experts’ opinions, translating them in an OHS context. This result is in line with the research hints provided by Lichtenberg et al. [41] and Damalas and Koutroubas [29]. To the authors’ knowledge this represents a novelty in context of the OHS literature. 

The proposed algorithm constitutes a basis for a simplified risk assessment procedure for the pesticides’ use and its implementation in a knowledge management system for risk assessment activities [86] is currently being considered, as well as its implementation in an OHS management system.

In addition, the current paper also aims at increasing the knowledge on the safe use pesticides. Such a result, in line with the practical needs highlighted by Rijal et al. [87], provides a general framework that integrates different disciplines and stakeholders. 

Moreover, although the proposed approach is based on the requirements of the Italian legislation, its general framework could be extended to other national contexts easily. As a matter of fact, the preliminary characterization of exposure levels scenarios allows safety managers to better define the potential damage and the evolution of hazard’s scenarios [88], supporting both the entrepreneur and the appointed physicians in effectively perform the workers’ health surveillance (WHS) that is mandatory in an OHS context in the EU countries [89]. 

Thanks to its simplicity and its task-based approach, the proposed methodology can be used also as a reference framework to augment the operators’ knowledge and awareness on a safe behavior, allowing the increase of all safety aspects within the company [90].

Beside these positive aspects, the study limitations have to be addressed as well. From a practical point of view, we have to consider that the selection of the types of toxicity listed in Table 9 cannot be considered exhaustive and a further analysis of possible toxicity typologies that can be found in the pesticides available on the market nowadays should be carried out. The whole approach of using hazard statements as a proxy of toxicity of substances needs validation, but any improvement in this aspect would easily be implemented in the proposed method. It has to be noted that in the present study the hazard statements related to the toxicity for the environment were not taken into account. For instance, for both product “A” and “B” the statement H400 (very toxic to aquatic life) was listed in the safety sheets. Since environmental concerns were not an objective of this study, an augmented approach considering also these issues could be beneficial for a more holistic risk assessment. In such a context, also the equipment’s cleaning operations should be included. 

Furthermore, we also need to underline that improved solutions such as the so-called mass trapping systems and bait sprays for the prevention of the olive fly were not considered in the study, since the proper use of such solutions to define the reduction of pesticide use quantitatively requires an expert’s analysis, which was not available when the case study was carried out. Nevertheless, we are aware that the implementation and promotion of eco-friendlier solutions to replace the use of hazardous substances is an important issue that needs to be investigated largely, as stressed by Damalas and Koutroubas [91].

From a scientific perspective, the proposed approach needs validation, which should be carried out by the parallel use of the proposed approach and environmental or biological monitoring to verify the effectiveness of the proposed factors and weights. This can be beneficial for a more accurate definition of the different weights’ ranges used for the computation of the various factors. Finally, the type of results achieved through a single case-study as a research tool can be considered exploratory and used to define new research questions and new understandings [92,93], but the findings’ external validity is limited by the sample concerned [94]. Accordingly, the application to different case studies concerning other types of cultivations and hence of pesticides is necessary.

## 7. Conclusions

The paper illustrates a procedure for the risk assessment of activities related to the application of pesticides, addressing the needs of farmers in complying with the recent legislative issues. This approach is based on studies concerning the application of the AHS algorithm and the related literature review [54,64,73]; through the integration of their findings with the provisions of technical guidelines and experts’ opinion, a preliminary risk assessment framework that can be used at a practical level to comply with the OHS legislation was defined. 

The merit of this paper consists in the overall development of a tool for the preliminary risk assessment, providing a simplified approach for its practical application. Our algorithm aims at evaluating all the parameters that might influence the exposure to pesticides, providing a qualitative result thorough the evaluation of a realistic situation. This supports the findings of Acquavella et al. [95], highlighting the limitations of the risk assessment approaches based on passive dosimetry in evaluating individual exposure situations effectively.

This study is at an initial step of implementation and the validation of the proposed approach through biological monitoring is already planned. Thus, researchers and practitioners are also invited to contribute to its further development.

## Figures and Tables

**Table 1:** EU legislation, its implementation in the Italian legislation, the context, and the related main issues.

Main Legislative Source	Italian Legislation	Context	Main Issues
Directive 89/391/EEC	Decree 81/2008	Occupational health and safety (OHS) of professional workers	Specific risk assessment of working activities related to the use of chemical products; Specific information and training of the operators that use pesticides; Provision of safe working equipment and specific personal protective equipment (PPE).
Directive 2009/128/EC	Decree 150/2012 and National Action Plan (NAP)	Sustainable use of pesticides	Certified information and training of the users, distributors, and advisors; Certified inspection of pesticide application equipment in professional use; Documented regular calibrations and technical checks of the pesticide application equipment; Proper handling and storage of pesticides and treatment of their packaging and remnants; Implementation of practices and products allowing low pesticide-input pest management.
Directive 2006/42/EC	Decree 17/2010	Machinery safety	Specific essential health and safety requirements for the construction and maintenance of machinery for pesticide application.
Regulation (EC) no. 1272/2008		Classification, labelling, and packaging of substances and mixtures	New type of classification and labelling of hazardous substances.
Regulation (EC) no. 1907/2006		Registration, evaluation, authorization and restriction of chemicals	New requirements for safety data sheets (containing information concerning hazards, first-aid measures, handling and storage, personal protection, etc.)

Notes: (1) In the table, only the main legislative acts are mentioned, while their further updates/amendments were not cited: e.g. concerning Directive 2006/42/EC, Directive 2009/127/EC was not mentioned. Similarly, when citing Directive 89/391/EEC we intend its consolidated text, including for example Directive 2009/39/EC. (2) Regulation (EC) no. 1907/2006 and its amendments (the so-called “REACH”) have not had a strong impact on pesticide users; it was included in the table due to the new rules on safety data sheets.

**Table 2:** Criteria used in the algorithm proposed by the Agricultural Health Study (AHS).

Factors	Criteria
MIX	Number of loadings; concentration of the active principle; type of formulation; duration of mixing and loading
APPL	Use rate; application pressure; treated area; interventions on machines during application; condition of the equipment; duration of the application
REPAIR	Maintenance of the equipment; duration of the maintenance

**Table 3:** Exposure factors during mixing and loading activities.

MIX = (LOAD × CONC) × COMP		
LOAD	Number of loads per day	Score
	1	0.5
	2–5	1
	>5	2
CONC	Concentration of the active principle	Score
	<50%	0.5
	50–90%	1
	>90%	2
COMP	Type of formulation/compound	Score
	Soluble bags	0.5
	Granules/liquid	1
	Powder	2

MIX: mixing; LOAD: loads; CONC: concentration; COMP: compound.

**Table 4:** Exposure factors during the application activities.

APPL = [(DOSE × SURF × BAR) ×TRACT] + INT + EQUIP		
DOSE	Quantity of pesticide used (kg/ha)	Score
	<0.1	1
	0.1–2.5	2
	>2.5	3
SURF	Application surface (ha)	Score
	<3	0.5
	3–10	1
	10–20	2
	>20	3
BAR	Application pressure/type of equipment (bar)	Score
	<3	1
	3–5	2
	5–10	3
	>10	4
TRACT	Type of tractor used to operate the pesticide application equipment	Score
	Without cabin	3
	With a non air-conditioned cabin	2
	With an air-conditioned cabin	1
	With an air-conditioned cabin equipped with activated carbon filters	0
INT	Number of in-field interventions to calibrate the equipment	Score
	0	0
	1–2	1
	>2	2
EQUIP	Condition of the application equipment	Score
	Good	0
	Not good	8

APPL: application; DOSE: dose; SURF: surface; BAR: pressure; TRACT: tractor; INT: interventions; EQUIP: equipment.

**Table 5:** Exposure factors during in-field maintenance of the application equipment.

**REPAIR**	**Equipment Maintenance Operations**	**Score**
	Maintenance operations are carried out by a different person	0
	Maintenance operations are carried out by the operator	30

**Table 6:** Exposure factors during re-entry activities.

RE-ENTRY = (DOSE × H/D) × EARL		
H/D	Plants height/foliage density	score
	Low/Low	1
	Low/High	2
	High/Low	3
	High/High	4
EARL	Time before re-entering in the field after the pesticide application in order to carry out other activities	score
	>2 days	0
	1–2 days (24–48 h)	1.5

**Table 7:** Exposure factors related to the condition of PPE, the operator’s experience, and the application frequency.

**PPEs**	**Condition of PPE**	**Score**
	Good	0.5
	Not good	1
**SKILL**	**Experience/Skills of the Operator in Using Pesticides**	**Score**
	>5 year	0.25
	1–5 years	0.5
	<1 year	1
**FREQ**	**Number of Days Per Year Dedicated to the Use of Pesticides**	**Score**
	<5	0.5
	5–10	1
	10–20	2
	>20	3

**Table 8:** Levels of possible exposure.

Exposure Level (I_exp_)	
Score	Meaning
≤5	Very low level of possible exposure
6–15	Low level of possible exposure
16–30	Medium level of possible exposure
31–50	High level of possible exposure
≥51	Very high level of possible exposure

**Table 9:** List of the toxicity levels based on Regulation (EC) no. 1272/2008.

Code	Hazard Statement	Toxicity Index (I_tox_)
H302	Harmful if swallowed	1
H319	Causes serious eye irritation	
H335	May cause respiratory irritation	
H315	Causes skin irritation	
EUH066	Repeated exposure may cause skin dryness or cracking	
H332	Harmful if inhaled	2
H312	Harmful in contact with skin	
H301	Toxic if swallowed	
H314	Causes severe skin burns and eye damage	
H318	Causes serious eye damage	
H331	Toxic if inhaled	3
H300	Toxic in contact with skin	
H317	May cause an allergic skin reaction	
H336	May cause drowsiness or dizziness	
H330	Fatal if inhaled	4
H310	Fatal in contact with skin	
H334	May cause allergy or asthma symptoms or breathing difficulties if inhaled	
H361f	Suspected of damaging fertility	
H360Fd	May damage fertility. May damage the unborn child	
H362	May cause harm to breast-fed children	
H304	May be fatal if swallowed and enters airways	
H371	May cause damage to organs	
H373	May cause damage to organs prolonged or repeated exposure	5
H370	Causes damage to organs	
H351	Suspected of causing cancer	
H372	Causes damage to organs through prolonged or repeated exposure	
H360F	May damage fertility	
H340	May cause genetic defects	
H360D	May damage the unborn child	

**Table 10:** Levels of the exposure risk (R_E_).

R_E_	Level	Meaning
≤15	I	Acceptable level of exposure risk
16–60	II	Medium level of exposure risk
61–150	III	High level of exposure risk
≥151	IV	Unacceptable level of exposure risk

**Table 11:** Improvement measures depending on the exposure risk’s level.

Exposure Risk Level (R_E_)	Improvement Measures
**Level I**	No additional interventions are required.
**Level II**	Update information and training activities; verify the adequacy and the condition of PPE. Verify the adequacy of the pesticides used.
**Level III**	Update information and training activities; verify the adequacy and the condition of PPE. Verify the adequacy of the pesticides used and the possibility of selecting less hazardous products, providing specific biological/environmental monitoring. Verify the adequacy and the condition of the pesticide application equipment and its maintenance operations. Update the operative procedures and instructions and carry out a new risk assessment.
**Level IV**	The adequacy of the pesticides used needs to be verified and the use of less hazardous products should be considered. A specific biological/environmental monitoring, as well as the operators’ health surveillance have to be implemented. Verify the selection of PPE and its condition as well as the adequacy of operative procedures and instructions. Verify the adequacy and the condition of the pesticide application equipment and its maintenance operations. Carry out a new risk assessment.

**Table 12:** Scores related to the calculation of the possible exposure level.

	Code		Product “A”	Product “B”
1. MIX	1.1	LOAD	1	1
	1.2	CONC	0.5	0.5
	1.3	COMP	1	1
	1.4	*t_M_*	0.042 (20 min)	0.042 (20 min)
2. APPL	2.1	DOSE	2	2
	2.2	SURF	0.5	0.5
	2.3	BAR	4	4
	2.4	TRACT	2	2
	2.5	INT	0	0
	2.6	EQUIP	0	0
	2.7	*t_A_*	0.312 (2.5 h)	0.312 (2.5 h)
3. REPAIR	3.1	REPAIR	30	30
	3.2	*t_R_*	0.125 (1 h)	0.125 (1 h)
4. REENTRY	2.1	DOSE	2	2
	4.1	H/D	3	3
	4.2	EARL	1.5	1.5
	4.3	*t_RE_*	0.042 (20 min)	0.042 (20 min)
5. PPE			0.5	0.5
6. SKIL			0.5	0.5
7. FREQ			1	2
I_exp_			2.036	4.073

**Table 13:** Evaluation of the Toxicity Index of both products.

Code	Hazard Statement	Toxicity Index (I_tox_)
**Product “A”**		
H302	Harmful if swallowed	1
H315	Causes skin irritation	1
H301	Toxic if swallowed	2
H318	Causes serious eye damage	2
H331	Toxic if inhaled	3
H317	May cause an allergic skin reaction	3
**Product “B”**		
H302	Harmful if swallowed	1
H319	Causes serious eye irritation	1
H315	Causes skin irritation	1
H317	May cause an allergic skin reaction	3
H330	Fatal if inhaled	4
H372	Causes damage to organs through prolonged or repeated exposure	5

**Table 14:** Scores related to the calculation of the exposure risk.

	I_exp_	I_tox_	R_E_	Level
**Product “A”**	2.0361	3	R_E_ = 6.11	I
**Product “B”**	4.073	5	R_E_ = 20.36	II

## Appendix A

The appendix contains an excerpt of the preliminary conformity checklist.
